# Cytotoxicity analysis of biomass combustion particles in human pulmonary alveolar epithelial cells on an air–liquid interface/dynamic culture platform

**DOI:** 10.1186/s12989-021-00426-x

**Published:** 2021-08-21

**Authors:** Shaorui Ke, Qi Liu, Xinlian Zhang, Yuhan Yao, Xudong Yang, Guodong Sui

**Affiliations:** 1 Co-construction Collaborative Innovation Center for Chinese Medicine and Respiratory Diseases by Henan & Education Ministry of P.R. China, Academy of Chinese Medical Sciences, Henan University of Chinese Medicine, Zhengzhou, 450046 People’s Republic of China; 2 Shanghai Key Laboratory of Atmospheric Particle Pollution Prevention (LAP3), Department of Environmental Science & Engineering, Fudan University, Shanghai, 200433 People’s Republic of China; 3 Department of Building Science, Tsinghua University, Beijing, 100084 People’s Republic of China; 4 Jiangsu Collaborative Innovation Center of Atmospheric Environment and Equipment Technology (CICAEET), Nanjing University of Information Science & Technology, Nanjing, 210044 People’s Republic of China

**Keywords:** Biomass combustion, Particles, HPAEpiC, Air–liquid interface, Dynamic culture, Cytotoxicity

## Abstract

**Background:**

Exposure to indoor air pollution from solid fuel combustion is associated with lung diseases and cancer. This study investigated the cytotoxicity and molecular mechanisms of biomass combustion-derived particles in human pulmonary alveolar epithelial cells (HPAEpiC) using a platform that combines air–liquid interface (ALI) and dynamic culture (DC) systems.

**Methods:**

HPAEpiC were cultured on the surface of polycarbonate (PC) membranes on the ALI–DC platform. The cells were sprayed with an aerosolized solution of biomass combustion soluble constituents (BCSCs) and simultaneously nourished with culture medium flowing beneath the permeable PC membranes. The ALI–DC method was compared with the traditional submerged culture approach. BCSC particle morphology and dosages deposited on the chip were determined for particle characterization. Flow cytometry, scanning electron microscopy, and transmission electron microscopy were used to investigate the apoptosis rate of HPAEpiC and changes in the cell ultrastructure induced by BCSCs. Additionally, the underlying apoptotic pathway was examined by determining the protein expression levels by western blotting.

**Results:**

Scanning electron microscope images demonstrated that the sample processing and delivering approach of the ALI–DC platform were suitable for pollutant exposure. Compared with the submerged culture method, a significant decline in cell viability and increase in apoptosis rate was observed after BCSC exposure on the ALI–DC platform, indicating that the ALI–DC platform is a more sensitive system for investigating cytotoxicity of indoor air pollutants in lung cells. The morphology and ultrastructure of the cells were damaged after exposure to BCSCs, and the p53 pathway was activated. The Bcl-2/Bax ratio was reduced, upregulating caspase-9 and caspase-3 expression and subsequently inducing apoptosis of HPAEpiC. The addition of *N*-acetyl cysteine antioxidant significantly alleviated the cytotoxicity induced by BCSCs.

**Conclusion:**

A novel ALI–DC platform was developed to study the cytotoxicity of air pollutants on lung cells. Using the platform, we demonstrated that BCSCs could damage the mitochondria, produce reactive oxygen species, and activate p53 in HPAEpiC, ultimately inducing apoptosis.

**Supplementary Information:**

The online version contains supplementary material available at 10.1186/s12989-021-00426-x.

## Background

Indoor air pollution is the fourth leading cause of death worldwide after hypertension, tobacco smoking, and alcohol consumption [1]. Solid fuel combustion is a major source of indoor air pollution [2, 3]; approximately 3 billion people depend on solid fuels, such as wood, charcoal, or other traditional biomass, to meet daily energy needs [4]. Moreover, diseases attributable to indoor air pollution from solid fuel combustion account for over 4 million premature deaths annually [5]. Solid fuel combustion produces high levels of hazardous indoor air pollutants, including respirable particulate matter, noxious gases, and other toxic chemicals [6]. Additionally, airborne particulates with large surface areas and strong adsorption capacities can harbor harmful bacteria, viruses, organic compounds, and heavy metals, which aggravate the severity of effects on cardiovascular and respiratory system health [7].

The lungs provide a critical barrier against direct exposure but are also the organ most severely damaged by indoor air pollution. Long-term exposure to indoor air pollution from solid fuel combustion has been shown to increase the occurrence and development of lung diseases [8]. A study has shown that 35.7% of respiratory diseases, 22% of chronic lung diseases, and 15% of bronchial diseases are associated with indoor air pollution [9]. Epidemiological statistics have also demonstrated a higher incidence of lung cancer among women in China than among those in several European countries with a higher prevalence of female smokers [10, 11]. This discrepancy may be related to the more widespread use of solid fuel stoves in China. Thus, investigating the effects of indoor air pollution from solid fuel combustion on lung health is essential for improving health outcomes.

Ultrafine particles (< 2.5-μm diameter) can directly enter pulmonary alveoli, damaging the respiratory system and suppressing immune function, and potentially inducing diseases such as pneumonia, emphysema, silicosis, and lung cancer [12, 13]. Heavy metals and organic compounds in airborne particles contribute to the pathogenesis of lung diseases via reactive oxygen species (ROS) generation and activation of related signal transduction pathways [14]. Lipophilic and low-molecular-weight compounds (< 1 kD) in airborne particles may bind to extracellular or membrane proteins, initiating signaling pathways that regulate cellular processes, such as proliferation, differentiation, and apoptosis. Previous studies have confirmed that fine particulate matter (PM_2.5_) from outdoor air pollution can stimulate intracellular ROS, activating the p53 signaling pathway and downstream protein expression, ultimately inducing inflammatory responses and apoptosis in human umbilical vein endothelial cells and lung epithelial A549 cells [15]. ROS generation produces oxidative stress, which can cause irreversible damage at the molecular and cellular levels. Lung cell inflammatory responses can trigger breathing problems, reduce lung function, and lead to lung diseases [16]. Therefore, elucidating molecular-level lung cell responses to airborne particles generated from solid fuel combustion is important for understanding health risks.

Recent studies have examined the cytotoxicity and molecular mechanisms of solid fuel combustion particles in lung cells [17, 18]. However, in vitro cytotoxicity analyses were generally performed using cells cultured under traditional submerged conditions, wherein particles are suspended in the culture medium containing submerged cells. Submerged exposures provide an unviable synthetic environment for the culture of lung cells, as they are entirely covered with the cell culture medium [19]. Alternatively, the air–liquid interface (ALI) technique can be used to achieve direct contact between aerosols and target cells that are cultured under a thin liquid film. This approach prevents the interference of the cell culture medium and maximizes the exposure area between the aerosol substance and the cells [19]. Consequently, cytotoxicity tests conducted under ALI exposure conditions provide more reliable reference values with practical applications. The ALI technique is, thus, the preferred culture method for in vitro toxicity testing of airborne particles with respect to pulmonary health.

Many particle-toxicity studies have adopted ALI systems to obtain new insights that could not be achieved using traditional culture methods [17, 20]. Some ALI studies have yielded results that are markedly different from those obtained using submerged culture methods. For example, nickel oxide and zinc oxide particles exhibited higher toxicities under ALI conditions than under submerged conditions [21]. Conversely, lower toxicities to lung cells were observed when silica nanoparticles and silver nanoparticles were cultured under ALI conditions compared with those obtained using submerged exposures [22]. Moreover, several studies have demonstrated that in vitro ALI culture tests can provide more reliable reference values than traditional methods [23].

The cell growth microenvironment is another factor that affects toxicity test results. The fluid shear produced in dynamic culture (DC) methods plays an important role in cellular function, development, differentiation, and metabolite secretion [24]. DC conditions can, for example, influence epithelial and endothelial cell behavior and significantly promote surfactant protein activity [25]. Moreover, DC techniques provide a dynamic flow of nutrients and metabolites, leading to improved intracellular metabolism and communication in vitro [24]*.* Thus, combining DC and ALI technology in vitro could provide a sensitive and reliable method for exploring lung cell molecular responses to toxic airborne particles that better mimics in vivo conditions.

**Fig. 1:**
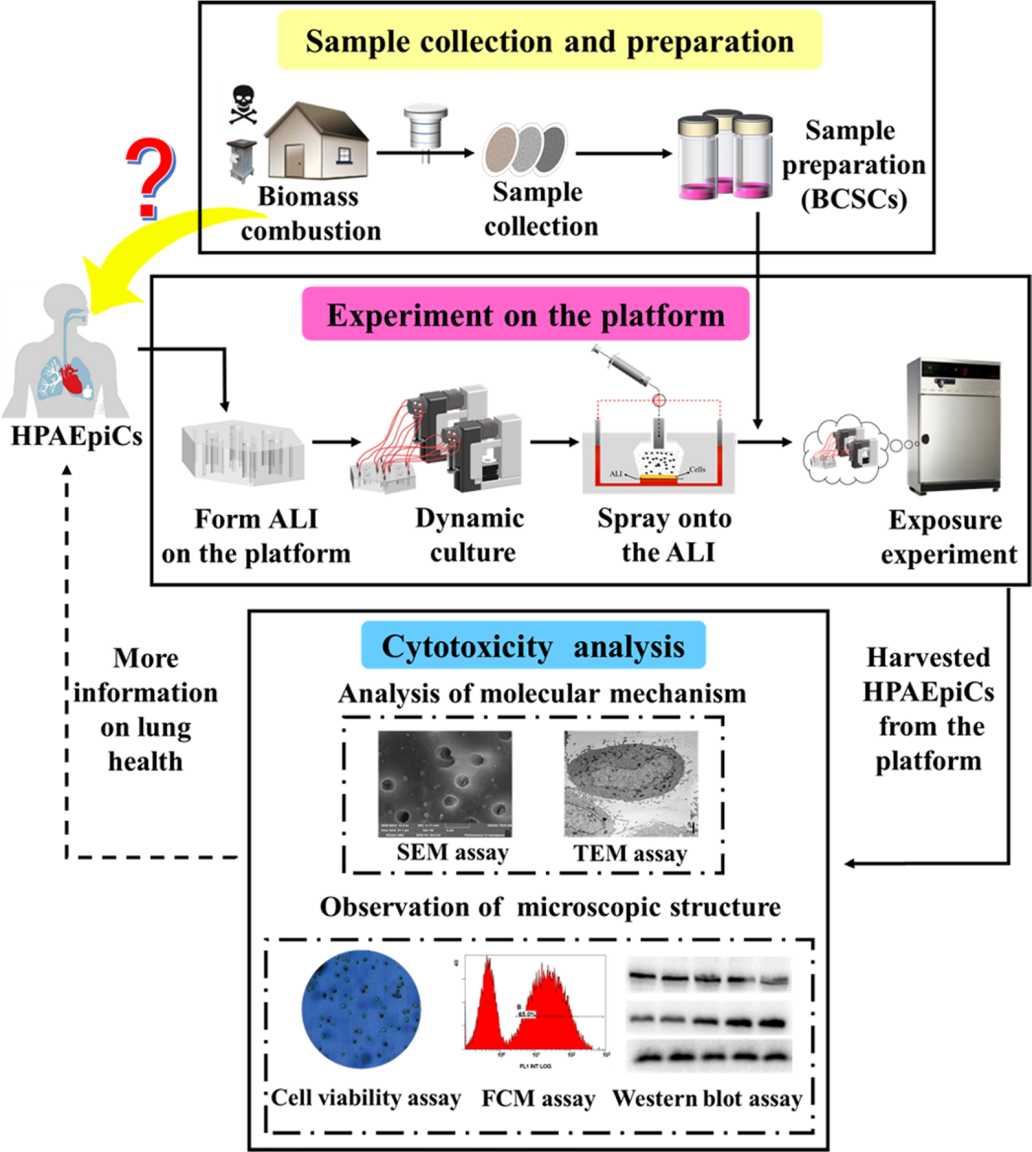
Study flow diagram for investigating the cytotoxicity and molecular mechanism of indoor air pollutants generated from biomass combustion in human pulmonary alveolar epithelial cells using a platform that combines air–liquid interface and dynamic culture systems

In this study, we developed a novel platform to simultaneously achieve ALI exposure and DC conditions to analyze the cytotoxicity and molecular mechanisms of biomass combustion particles in human pulmonary alveolar epithelial cells (HPAEpiC). HPAEpiC were cultured on the surface of 3-μm pore polycarbonate (PC) membranes, and the apical surface was exposed to aerosol particles. The cells were nourished with a medium flowing beneath the membranes in the platform, combining ALI exposure and DC conditions in vitro. The objectives of this study were to gain a comprehensive understanding of the cytotoxic effects and potential molecular mechanisms of biomass combustion soluble constituents (BCSCs) in HPAEpiC and assess potential risks to public health associated with BCSCs (Fig. 1).

## Results

### ALI–DC platform fabrication and cell cultivation

**Fig. 2:**
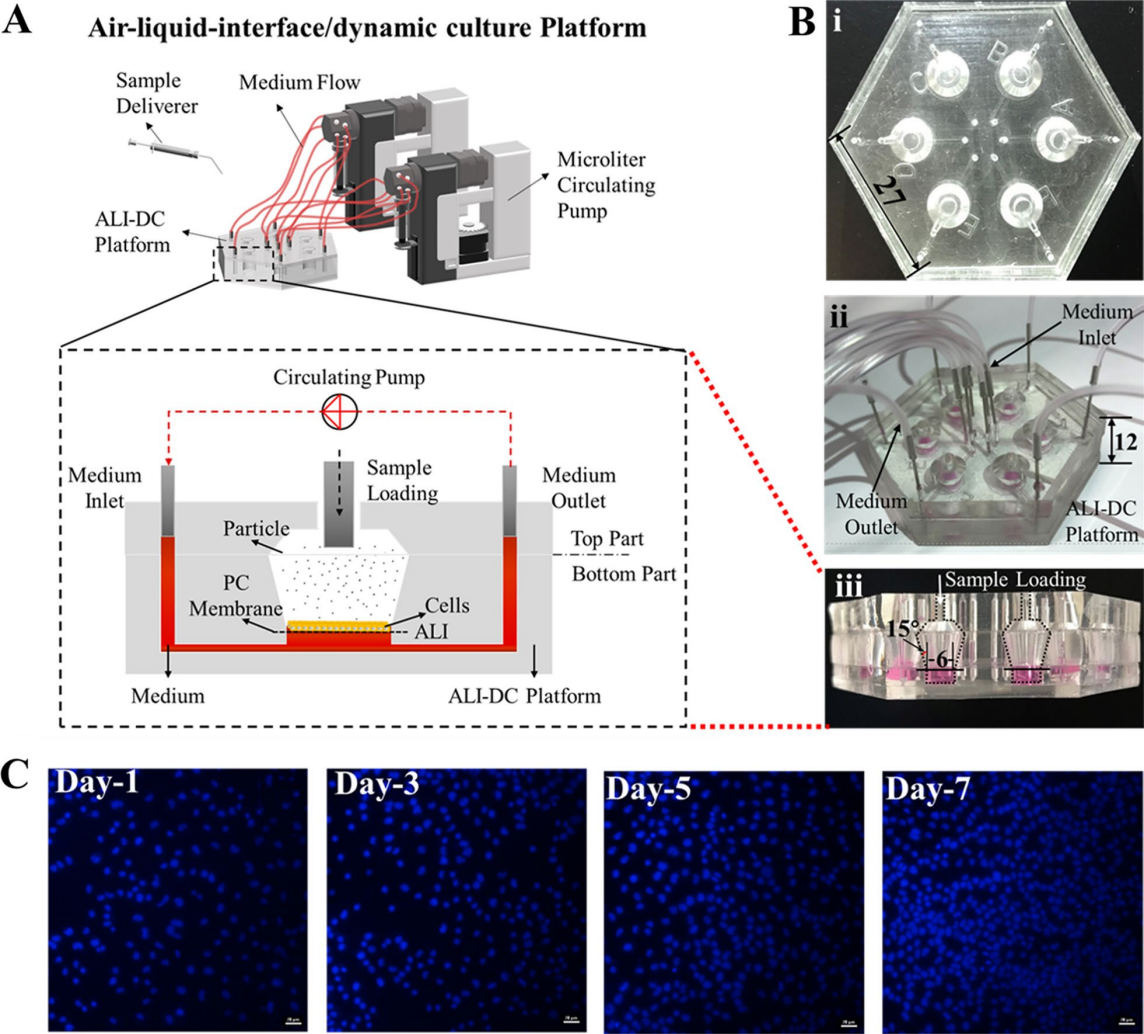
**A** A schematic illustration showing the air–liquid interface (ALI)-dynamic culture (DC) platform and ALI exposure concepts. **B** Photographs of the fabricated ALI–DC platform (i), experimental setup for DC in the platform (ii), and basic structure of the platform for cellular exposure to test substances (iii), length unit: mm. **C** Photographs of DAPI-stained HPAEpiC nuclei cultured on the modified polycarbonate membrane on days 1, 3, 5, and 7, respectively. Scale bars = 20 μm

The ALI–DC platform was fabricated to achieve simultaneous ALI exposure and DC conditions to study the cytotoxicity of air pollution on lung cells. The hexagonal (side length, 27 mm; height, 12 mm) microfluidic chip consists of six repetitive units, each containing a circular PC membrane approximately 9 mm in diameter (Fig. 2B-i). The PC membrane is placed on top of a 6-mm-wide circular hole connected to two thin channels (0.6-mm wide, 0.1-mm high). Above the PC membrane is a tapered chamber with a 6-mm base diameter at a 15-degree inclination (Fig. 2B-ii, and iii).

Cell growth was observed using 4′,6-diamidino-2-phenylindole (DAPI) in-situ staining to verify whether the PC membrane was successfully modified and suitable for cell adhesion. The cell density gradually increased as the culture time progressed, reaching 80% on day five and exceeding 90% on day seven, indicating that the cells were successfully proliferating (Fig. 2C).

### Particle deposition on the platform

**Fig. 3:**
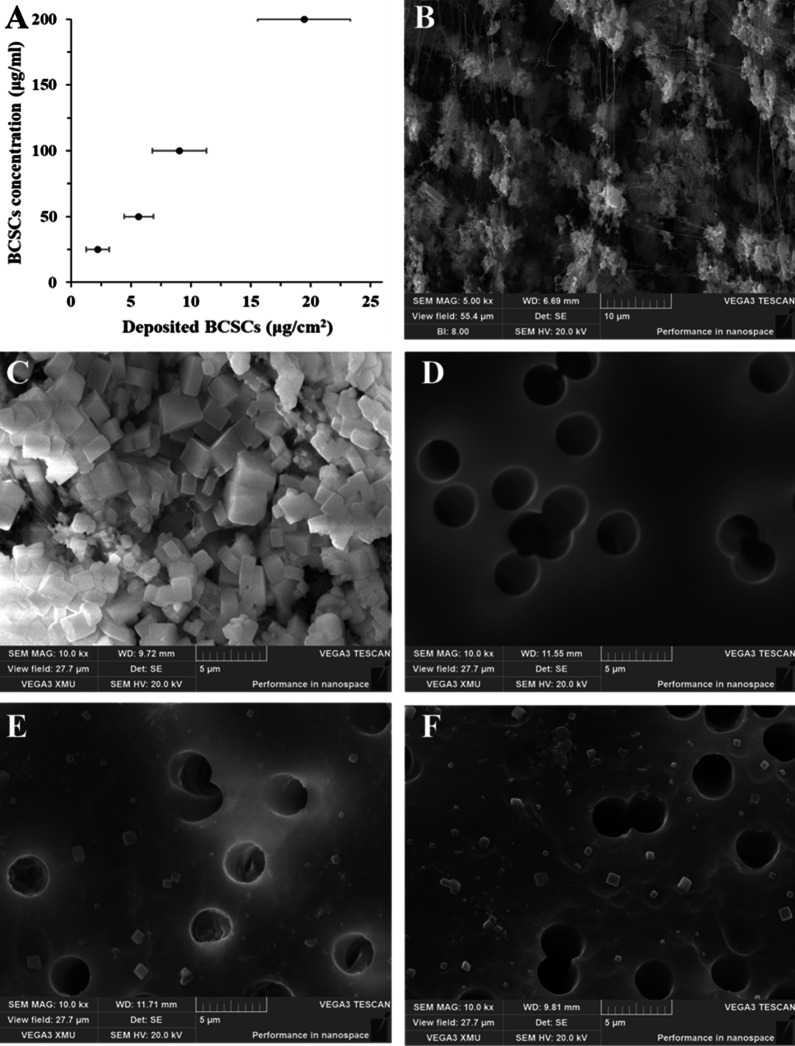
**A** Average deposited doses (μg/cm^2^) of biomass combustion soluble constituent (BCSC) samples; **B** Scanning electron microscope (SEM) image of Teflon membrane; **C** SEM image of particles collected on the Teflon membrane. **D** SEM image of polycarbonate (PC) membrane; **E** and **F** SEM images of PC membranes exposed to 100- and 200-μg/ml BCSC samples, respectively

The average deposited doses on the membrane (Fig. 3A) were 2.23 ± 0.93, 6.21 ± 1.94, 13.76 ± 3.48, and 18.19 ± 4.32 μg/cm^2^, corresponding to the atomized samples of 25, 50, 100, and 200 μg/ml BCSCs, respectively. As shown in Scanning electron microscope (SEM) images in Fig. 3B, C, the irregular polygonal particles were smaller than 5 μm in diameter, and most were less than 2.5 μm, categorizing them as fine particles. Moreover, Fig. 3E, F show that the microscopic appearance (shape and size) of the BCSC particles deposited on the PC membrane of the chip was similar to those deposited on the Teflon sampling membrane (Fig. 3C).

### Comparison of the ALI–DC platform and submerged culture conditions

**Fig. 4:**
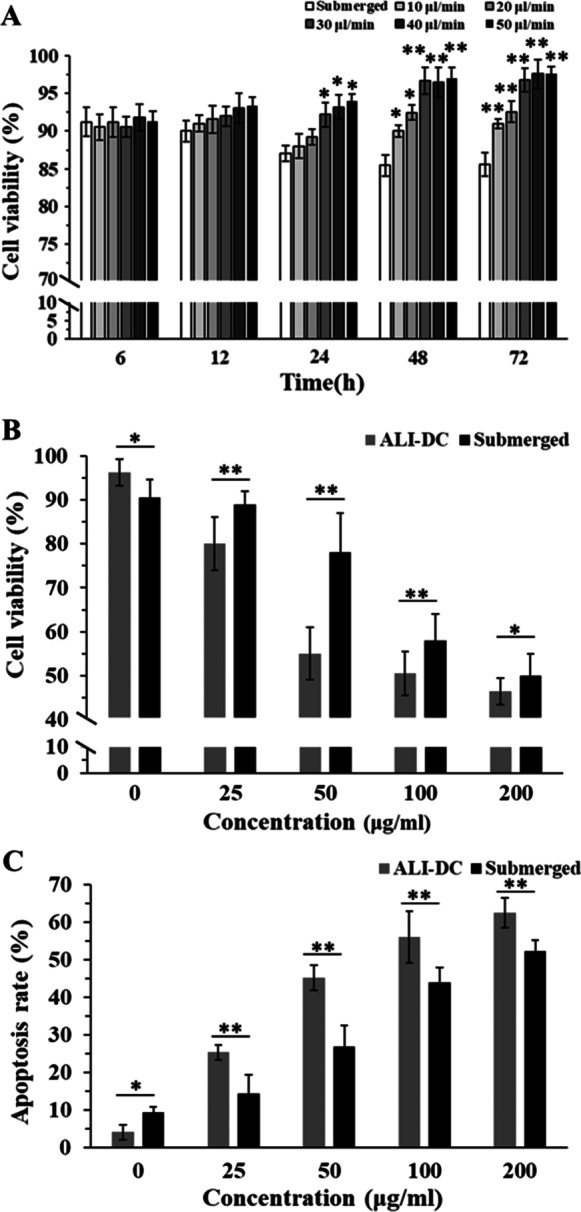
**A** Comparison of HPAEpiC viability under submerged conditions and air–liquid interface (ALI) and dynamic culture (DC) conditions with different medium flow rates; **B** HPAEpiC cell viabilities and **C** apoptosis rates after 24 h exposures to 0, 25, 50, 100, and 200 μg/ml biomass combustion soluble constituents (BCSCs). (*P < 0.05, **P < 0.01)

We cultured HPAEpiC under submerged conditions and ALI–DC conditions with different flow rates to optimize our platform. As the cultivation time progressed, cell viability in the submerged culture conditions decreased from 91.4 to 85.4% (Fig. 4A). Comparing the ALI–DC platform flow rates, the cell viabilities at flow rates from 10 to 50 μl/min did not show apparent differences at 6 and 12 h. At 24 h, the cell viabilities gradually increased with increasing flow rates, and the cell viabilities at flow rates of 30 μl/min and higher were significantly different from those in the submerged conditions. The cell viabilities at flow rates of 30, 40, and 50 μl/min were not significantly different from each other. Thus, a flow rate of 30 μl/min was selected for culturing cells on the ALI–DC platform for 24 h.

We exposed HPAEpiC to 0, 25, 50, 100, 2000 μg/ml BCSCs under submerged conditions and in the ALI–DC platform with a flow rate of 30 μl/min. In the groups treated with 0 μg/ml BCSCs (control), the cell viability was significantly higher in the ALI–DC platform (96.2%) than in the submerged culture (90.6%), and the apoptosis rate was significantly lower in the ALI–DC platform (4.37%) than in the submerged culture (9.84%) (Fig. 4B, C). However, after exposure to BCSCs (25–200 μg/ml), the cell viabilities in the ALI–DC condition were significantly lower than those in the submerged conditions. The difference in cell viability between the two methods was greatest when cells were exposed to 50 μg/ml BCSCs (Fig. 4B). In the BCSC exposures, cells cultured in the ALI–DC platform exhibited higher apoptosis rates than those under submerged conditions (Fig. 4C).

### HPAEpiC morphological changes

**Fig. 5:**
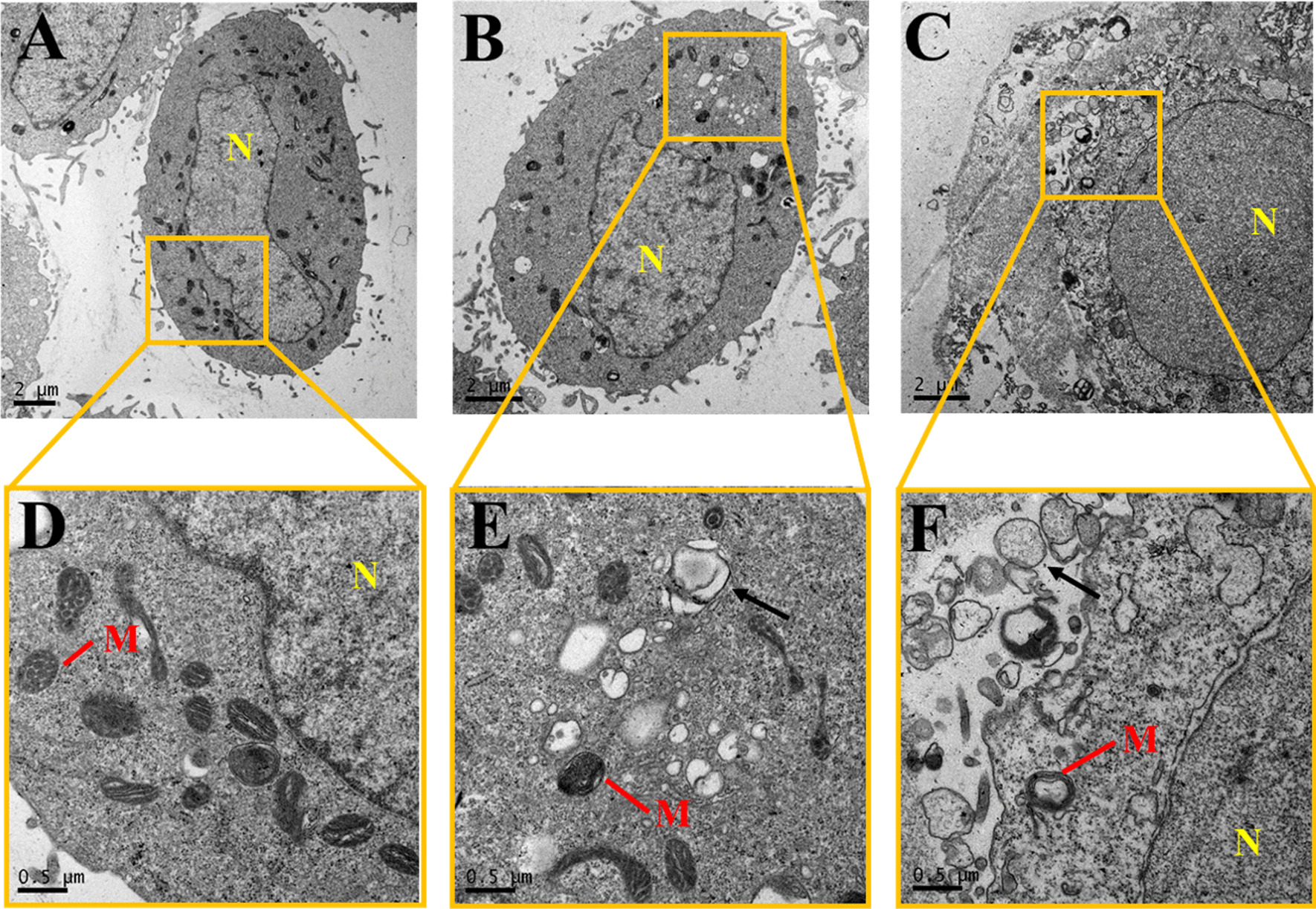
Transmission electron microscopy images of HPAEpiC in (**A**) and (**D**) the control group, and (**B**), (**C**), (**E**), and (**F**) in the experimental group exposed to 200 μg/ml biomass combustion soluble constituents. (N: nucleus; M: mitochondria; Black arrow: vacuoles)

Transmission electron microscopy (TEM) was used to compare the ultrastructure of HPAEpiC after treatment with 0 and 200 μg/ml BCSCs for 24 h. The TEM images showed that in the control group (0 μg/ml BCSCs), the contour of the cells was clear and smooth, the microstructure remained intact, and intracellular mitochondrion structures were well defined (Fig. 5A, D). However, in the group exposed to 200 μg/ml BCSCs, the cell morphology was severely damaged (Fig. 5B, C); changes in the cytoplasmic membrane, increased vacuolization in the cytoplasm, and degeneration of mitochondria were observed (Fig. 5E, F).

### Activation of p53, caspase-9, caspase-3 and decrease in the Bcl-2/Bax ratio

**Fig. 6:**
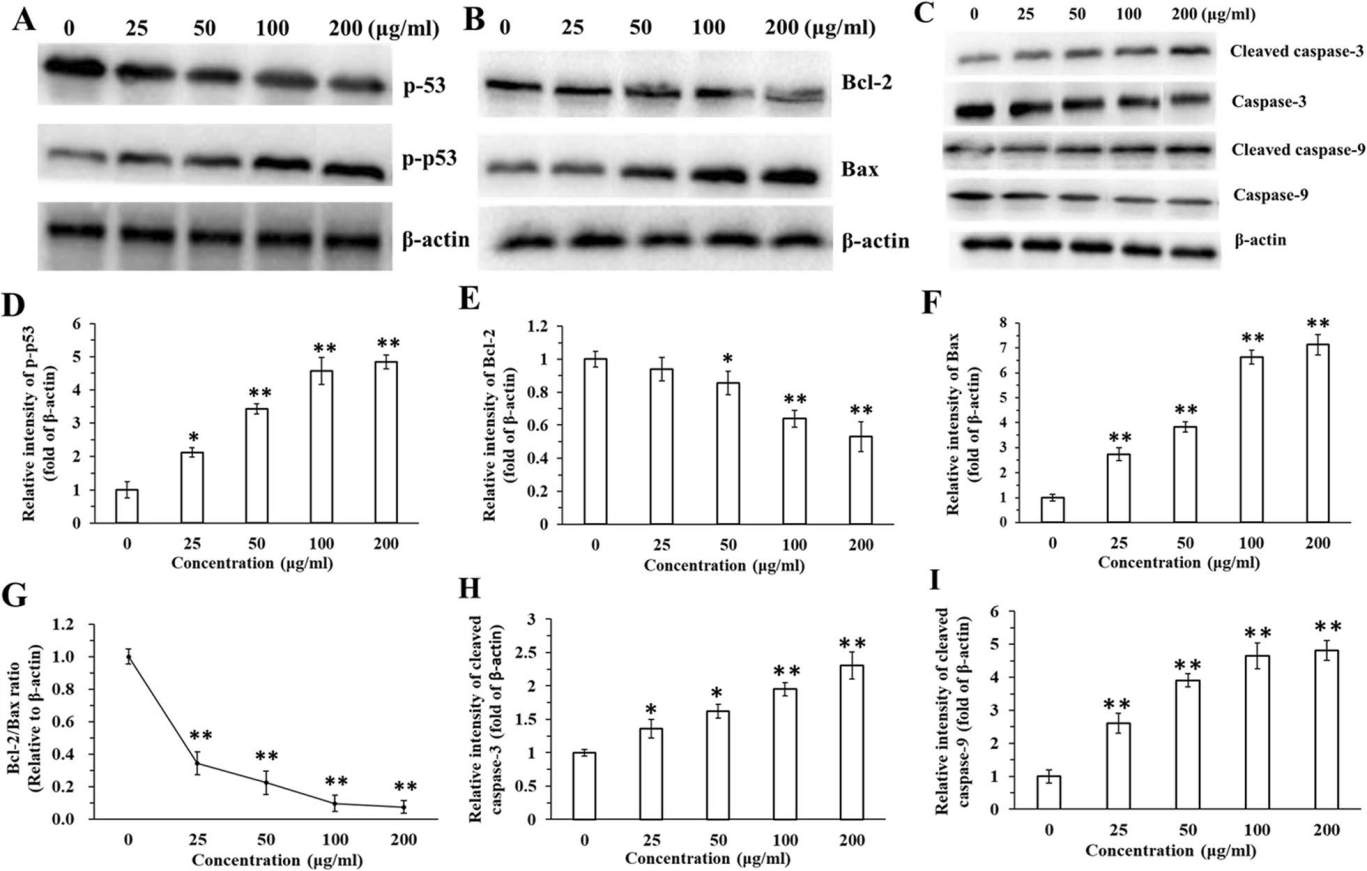
Representative Western blot analysis of **A** p-p53; **B** Bax and Bcl-2; and **C** cleaved caspase-3, caspase-3, cleaved caspase-9, and caspase-9, with β-actin as a loading control in human pulmonary alveolar epithelial cells exposed to 0–200 μg/ml biomass combustion soluble constituents (BCSCs). The relative gray value of **D** p-p53, **E** Bcl-2, **F** Bax, **G** Bcl-2/Bax, **H** cleaved caspase-3 and **I** cleaved caspase-9 in human pulmonary alveolar epithelial cells. (*P < 0.05, **P < 0.01)

Western blot analyses revealed that the expression of phosphorylated p53 increased with exposure to BCSCs (Fig. 6A, D). The results also showed that Bax expression levels significantly increased in a dose-dependent manner with increasing BCSC concentrations (Fig. 6B, F). Conversely, the anti-apoptotic Bcl-2 expression level decreased with increasing BCSC doses (Fig. 6B, E). Compared with the control (0 μg/ml BCSCs), the Bcl-2/Bax ratio was reduced by more than 60% in the 25 μg/ml BCSC group and continually decreased with increasing doses of BCSCs (Fig. 6G). The expression of caspase-9 and caspase-3 declined with increasing concentrations of BCSCs, while the expression of cleaved caspase-9 and -3 increased (Fig. 6C). The expression of cleaved caspase-9 and -3 both increased remarkably after exposure to BCSCs, even at the lowest dose (Fig. 6H, I).

## Discussion

### ALI–DC platform design and sample exposures

In our novel ALI–DC platform, HPAEpiC were cultured on the surface of PC membranes, exposed to BCSCs on the apical surface, and nourished with the culture medium flowing beneath the membranes. Compared with the traditional submerged culture method, cell viability was significantly higher and apoptosis rate was significantly lower under ALI–DC conditions (Fig. 4). The ALI–DC platform provides a simulated inhaled air environment for lung cells cultured on PC membranes and increases nutrient and catabolite turnover using dynamic culture. A previous study reported that under the flow conditions in a bioreactor, the cell viability, nutrient acquisition, and sensitivity of endothelial cells were all increased, making the cells more susceptible to the effects of silver nanoparticles; thus, increasing their cytotoxicity [26]. Similar promoting effects were observed in a dynamic bioreactor for cultivating human aortic endothelial cells and microfluidic devices for differentiation of mouse embryonic stem cells [27, 28]. The flow conditions in DC have been demonstrated to facilitate cellular metabolism. Therefore, the ALI–DC platform provides a suitable surface and active nourishment for the growth of HPAEpiC.

For the sample exposure on the ALI, we developed a tapered chamber to the platform for aerosol delivery. The traditional vertical cylindrical chamber above ALI typically creates a dead end on the edge of the cell substrate to prevent particle distribution on the ALI [29, 30]. As a result, the cells growing in the dead end are not exposed to particles, thereby affecting the experimental results. Our preliminary experiments confirmed that the ALI–DC chamber with a 15-degree inclination could inhibit the formation of exposed dead ends and allow the particles to become more evenly distributed on the PC membrane, thereby solving the abovementioned problem effectively. Thus, an ALI–DC chamber with a 15-degree inclination was considered beneficial. With the combination of the spray-type delivery and the tapered chamber, the aerosolization efficiency of the sample was between 42 and 64%, and the deposited doses were positively correlated with the BCSC concentrations (Fig. 3A). These findings demonstrate that a stable sample delivery approach was achieved on the platform. Moreover, no obvious effects of sample processing on the BCSC particle state were observed in the SEM images (Fig. 3B, C). We also performed ET toxicity tests to confirm that bacterial ET in the BCSC samples would not affect HPAEpiC growth (Additional file 1: Fig. S1). Our results show that the sample collection, extraction, and atomization methods used in our study were feasible. Therefore, the ALI–DC platform exposure is more effective than conventional methods for exploring lung cell molecular responses to toxic substances, making it practical for research on air pollution and drugs for pulmonary administration.

### Evaluation of the ALI–DC platform for BCSC toxicity tests

Gas exchange in the lung alveoli, where a layer of fluid covers each alveolar surface, forms a liquid–air interface [31]. Thus, ALI exposures provide a more practical and effective way to simulate the lung environment compared with traditional submerged exposures [23, 32, 33]. To validate the applicability of the ALI–DC platform, we compared the cytotoxic effects of BCSCs on cells cultured on the ALI–DC platform and under submerged conditions. Our results show that the BCSCs exerted higher cytotoxicity to HPAEpiC on the ALI–DC platform than under submerged conditions (Fig. 4). Similarly, a study assessing zinc oxide nanoparticle toxicity reported that lung epithelial A549 cells suffered more severe damage when exposed to ZnO nanoparticles under ALI conditions than under submerged conditions [34]. Moreover, vehicle exhaust pollutants also showed different cytotoxicity to lung epithelial BEAS-2B cells between the two culture methods [35]. In ALI exposures, the thin liquid film covering the cells provides a higher contact efficiency between the particles and the cells than that achieved using submerged culture methods [9, 36, 37]. Therefore, the ALI–DC platform is more effective and sensitive than conventional methods for exploring the molecular responses of lung cells to toxic substances.

### Cytotoxic effects of BCSCs on HPAEpiC

Particle size is an important factor related to the biotoxicity of air pollution. SEM images (Fig. 3C) show that the BCSC particles used in this study can be classified as fine particles (< 2.5 μm, PM_2.5_). Fine particles in indoor air can travel past the villi in the nose and trachea, reaching the alveoli of the lungs, where they can cause severe damage [4, 38]. Many studies have demonstrated that PM_2.5_ can cause cytotoxicity in human cells [39, 40]. In our previous study, we demonstrated that BCSCs could induce inflammatory responses and apoptosis in human keratinocytes. We observed similar BCSC toxicity in HPAEpiC, and the effects were dose-dependent (Fig. 4). BCSCs can contain a variety of toxic substances, including polycyclic aromatic hydrocarbons, volatile organic compounds, and heavy metals [41, 42]. These substances can induce cellular structural damage, functional or metabolic disorders, and apoptosis [43]. Clinical studies have shown that lung cell apoptosis is implicated in a wide range of pulmonary diseases, including pulmonary edema, acute lung injury, pulmonary fibrosis, and pneumonia [44, 45]. Even low concentrations of indoor air pollution from solid fuel combustion can increase the incidence of lung diseases.

TEM imaging revealed abnormal morphologies of mitochondria and other signs of cellular injury, further demonstrating the toxic effects of BCSCs on HPAEpiC (Fig. 5) and reflecting the cell viability and apoptosis rate results. Mitochondrial damage can result from ROS stress induced by PM_2.5_, which severely affects normal cellular metabolism. Previous studies showed that ROS generation triggered by PM_2.5_ produced significant decreases in the viability of EA.hy926 human endothelial cells and activated autophagy in A549 cells. [46, 47]. Understanding the molecular mechanisms of BCSC-mediated cytotoxicity is essential to provide an experimental basis for the prevention of lung injury and diseases. It is worth noting that the ultrasonic process we used to remove and sterilize particles may increase ROS production [48]. However, since all treatment groups were exposed to the same processes, the relative ROS production in the control and treatment groups still provides a valid comparison. In future studies, we can examine the ROS-generating capacities of sonication techniques to provide meaningful support for research on particulate matter.

### Molecular mechanisms underlying BCSCs-induced cytotoxicity in HPAEpiC

Fine particles can attack cells via oxidizing intercellular macromolecules (e.g., ROS), disrupting gene expression and signaling apoptosis pathways [49]. Our results showed that BCSCs significantly increased ROS production in HPAEpiC (Additional file 1: Fig. S2) and damaged mitochondria (Fig. 5)—the primary producers of ROS. It is well-known that increased ROS production can induce the phosphorylated p53 pathway and activate the expression of downstream proteins [50]. The target proteins in this apoptosis-related pathway are cancer-causing biological factors [51]. Our western blot analysis revealed that phosphorylated p53 expression was upregulated after exposure to BCSCs (Fig. 6A). Additionally, simultaneous exposure of HPAEpiC to NAC and BCSCs markedly decreased ROS generation and phosphorylated p53 expression. These findings suggest that the ROS-mediated p53 pathway is the primary pathway involved in BCSC-induced apoptosis in HPAEpiC.

**Fig. 7:**
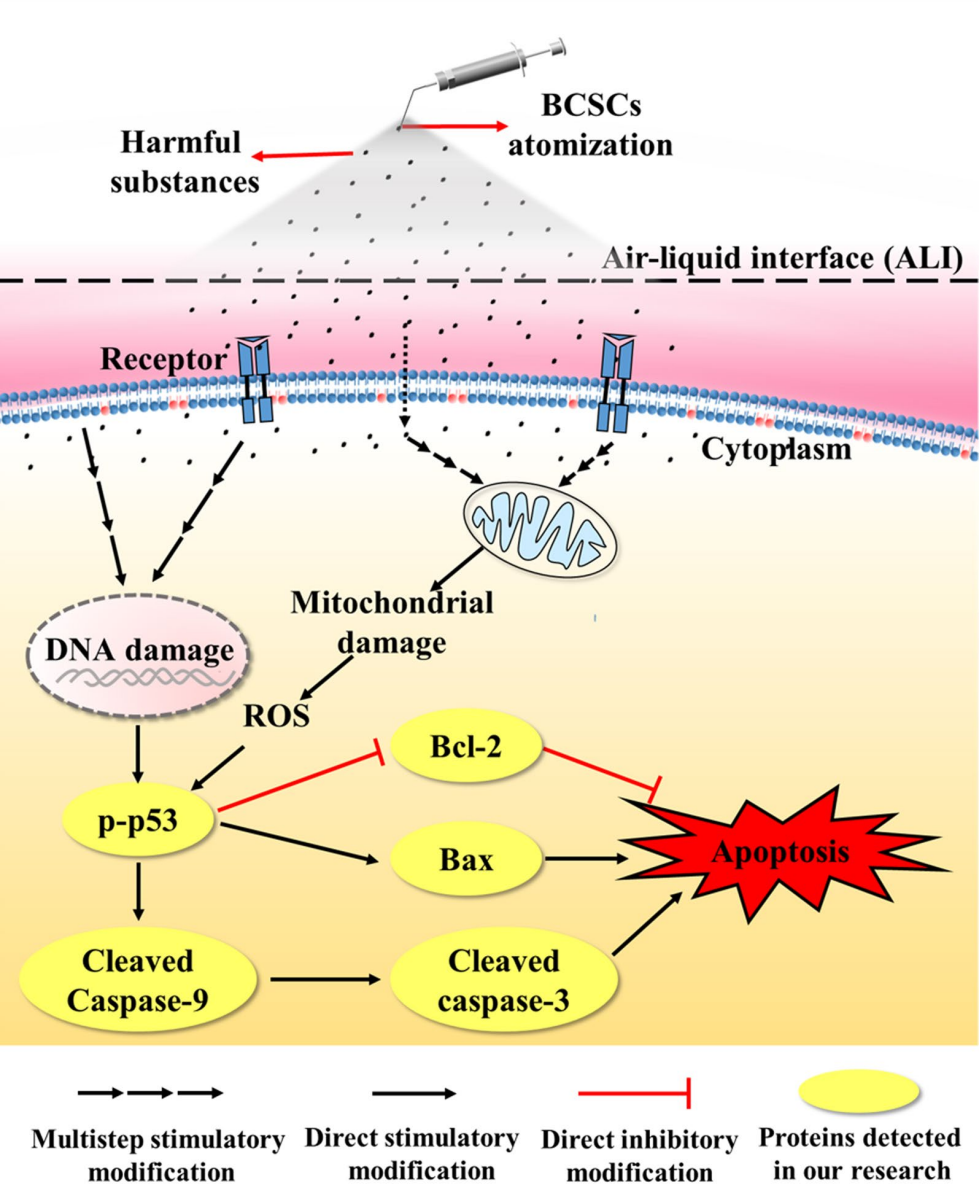
Schematic illustration of the molecular mechanism underlying of biomass combustion soluble constituent (BCSC)-induced cytotoxicity in human pulmonary alveolar epithelial cells (HPAEpiC). The p53 signaling pathway was activated after HPAEpiC exposure to BCSCs, significantly enhancing the expression of Bax, cleaved caspase-9, and cleaved caspase-3, and inhibiting Bcl-2 expression

The activation of p53 also regulates Bcl-2 protein family-related mitochondrial pathways; thus, we measured anti-apoptotic Bcl-2 protein and pro-apoptotic Bax protein expression [52]. The ratio between these two proteins reflects cell susceptibility to apoptosis [53]. We observed a decrease in the Bcl-2/Bax ratio in BCSC-treated HPAEpiC, providing further evidence that the p53 pathway is involved in apoptosis induced by BCSCs (Fig. 6). Furthermore, the expression of downstream protein caspase-9 and caspase-3 were activated, ultimately inducing apoptosis. In conclusion, our study demonstrated that BCSCs damage mitochondria, produce ROS, activate the p53-bcl-caspase pathway, and eventually induce apoptosis of HPAEpiC (Fig. 7).

However, it should be noted that after adding NAC, the apoptosis rates of the treatment groups were still significantly higher than that of the control group (Additional file 1: Fig. S3). This result suggests that BCSCs activate other apoptosis-related signaling pathways except the p53 pathway. Therefore, further research on the molecular mechanisms underlying BCSC cytotoxicity in lung cells is warranted.

## Conclusion

In this study, we fabricated a novel ALI–DC platform to investigate the cytotoxicity of BCSCs in HPAEpiC. The platform allows direct exposure of cells to aerosol test substances, while also providing circulating DC conditions. Compared with traditional submerged exposure methods, the ALI–DC platform is a more sensitive and effective approach for investigating the cytotoxicity of substances to lung cells, particularly for studies on air pollution and drugs for pulmonary administration. Our study demonstrates, for the first time, that BCSCs induce the apoptosis of HPAEpiC by activating the ROS–p53–caspase pathway. These findings offer theoretical evidence for biomass combustion-induced cytotoxicity in lung cells, lay a foundation for future studies on this topic, and may contribute to the prevention and treatment of lung diseases induced by indoor air pollutants from solid fuel combustion.

## Methods

### Biomass combustion sample collection and treatment

Biomass combustion experiments were conducted at the Tsinghua University in Beijing, China, in October 2016. The laboratory was 6.2 m long, 2.4 m wide, and 3 m high (approximate volume, 44.64 m^3^). All windows and doors were closed during the combustion experiments. A laboratory oven with an internal diameter of 15 cm was used for biomass combustion. Each combustion experiment consumed approximately 3 kg of corn stalk biomass. During the biomass combustion process, fine particles were collected on Teflon membranes using a high-capacity sampler (Thermo Fisher, MA, USA) placed at a height of 1.2 m and operated at a uniform flow rate of 3 L/min. Combustion experiments were repeated three times a day for 7 days of continuous sampling.

The membranes used for collecting samples were then cut into small pieces (approximately 1.5 × 1.5 cm^2^) and placed in a brown glass bottle. After adding 50 ml of double-distilled water, the bottles were treated using ultrasonic waves (D6, GT SONIC, China) for 30 min. Next, the suspension was dried using a vacuum freeze dryer. The dried membrane pieces were immersed in methanol and treated with ultrasonic waves for 2 h, after which the suspension was dried again as described above. Finally, we collected the powders in centrifuge tubes and stored the samples at − 20 °C until use. Powders were re-suspended in 1,000 μg/ml Dulbecco’s modified Eagle medium (DMEM) and sterilized by sonication for 30 min before use. Prior to the experiment, the stock solution was diluted in DMEM to final concentrations of 25, 50, 100, and 200 μg/ml BCSCs. In 0-μg/ml BCSC samples (the control group), we also cut empty membranes into small pieces and administered the same treatment as used in other groups. Finally, the control group was added with DMEM.

### ALI–DC platform design and fabrication

The ALI–DC platform was designed to achieve simultaneous exposure of HPAEpiC to BCSCs under ALI and DC conditions. The platform was fabricated with six repetitive units and separate top and bottom parts (Fig. 2A). The top part contains six inlets and six outlets, which were used for circulating the medium. Six sample loading holes were used to inject the BCSC solutions into the ALI system. Each sample loading hole was covered with permeable polydimethylsiloxane (PDMS) film to prevent cell contamination and volatilization of the medium. PDMS films were punctured for sample injection, after which the sample loading holes were covered with a new PDMS film. The bottom part contains six exposed chambers placed at 15-degree inclinations to achieve uniform particle distribution on the PC membranes that were placed beneath the holes, providing a substrate for cell growth (Fig. 2B-iii). Beneath each PC membrane was a chamber filled with the culture medium. Cells on the PC membrane surfaces were nourished with the medium flowing below the hydrophilic permeable membranes. The cells were exposed to air on the apical surface, forming an ALI. Additionally, each chamber had two channels that were connected to a circulating pump to provide DC conditions.

We designed the ALI–DC platform using CAD software and sent the pattern to the processing company (Yinmeng 9800, Shanghai Yinmeng Technologies Co., Ltd., China) to manufacture the copper mold. After receiving the copper mold, two types of PDMS prepolymer (RTV 615A and B in 5:1 and 7:1 ratios) were prepared and poured into the mold. We used a vacuum pump to remove bubbles before heating the copper mold in an oven at 80℃ for 1 h to solidify. The cured PDMS chip was then gently separated from the copper mold, and the chip was cut, punched, and assembled. The PC membranes were placed on the PDMS layer under a microscope, and the platform layers were aligned and dried at 80℃ for 1 h to prevent chamber leakage. The ALI–DC platform was sterilized at 121 °C for 30 min and dried at 60 °C prior to use.

### Aerosolization efficiency and deposited doses on the ALI–DC platform

PC membranes were weighed using an ultramicro electronic balance (Maitre Toledo Electronic Scale, XPR6U) before assembling onto the ALI–DC platform (*m*_0_). The membranes were sandwiched into the designated PDMS parts (but not bonded to facilitate membrane weighing) to achieve an efficient deposited dose using the chambers with 15-degree inclinations. We used a high-pressure syringe (Penn-Century, USA) to atomize the sample, drawing a portion of the air and 10 μl of the sample into the syringe. For atomization, all of the air was expelled from the syringe, which ensured the atomization of the 10-μl volume. Ten-microliter aliquots of BCSC solutions of each concentration were atomized into the chambers six times at 20-min intervals (total sample delivery volume, 60 μl). Sample delivery was conducted 10 times to obtain a deposited dose that could be detected using the ultramicro electronic balance. Next, the membranes were carefully removed from the platform and dried in a vacuum freeze dryer. Finally, the membranes carrying deposited particles were weighed again (*m*_total_). The average deposited dose ($\overline{\mathrm{D} }$) and aerosolization efficiency ($\overline{\mathrm{E} }$) were calculated using the following formulas (Eq. 1–3):

1 $$\overline{{m }_{d}}=\frac{1}{3}\sum \left({m}_{total, i}-{m}_{0, i}\right)/10$$

2 $$\overline{\mathrm{D} }=\overline{{m }_{d}}/S$$

3 $$\overline{\mathrm{E} }=\overline{{m }_{d}}/{m}_{a}$$

where $\overline{{m }_{d}}$ is the average mass of particles deposited on the PC membrane, where *i* is the number of sample delivery (*i* = 1, 2, or 3), *S* is the cell growth area in each chamber of the ALI–DC platform (*S* = 0.28 cm^2^), ${m}_{a}$ is the total mass of atomized particles, calculated as BCSC concentrations × atomization volume (600 μl).

### Cell culture and BCSC exposure on the ALI–DC platform

Poly-l-lysine can promote cell adhesion and adherence; thus, it is commonly used as a cell adhesion agent in cell cultures. PC membranes were soaked with 0.01% poly-l-lysine solution for 8 h to encourage the adherence and growth of cells in a dynamic cell culture. The medium was slowly injected into the chambers beneath the membrane via the bottom inlets prior to cell seeding. Subsequently, 100 μl of the cell solution (1 × 10^6^ cells/ml) was seeded on the upper surface of each membrane (S = 0.28 cm^2^). HpAEpiC cells were cultured in a high-glucose DMEM containing 10% fetal bovine serum and penicillin/streptomycin (100 units/ml) and incubated at 37 °C under 5% CO_2_ and 95% humidity. After static cultivation for 2 h, the medium above the membrane was removed, forming an ALI on the upper surface of the PC membrane. To create DC conditions in the thermostatic incubator, the bottom part of the chip was covered with the top part, and each sample loading hole was covered with a PDMS film. Two circulating pumps (Hamilton, PSD/4 syringe pump) were used to supply the medium flowing on the chip.

Because the cells cultured on the membrane could not be observed directly using bright-field microscopy, the growth density was measured using a cell counting plate (cell density in the cell suspension/mL = total number of 4 large cells/4 × 10 000 × 10). Two parallel groups were performed in each experiment, one for the cell density measurements and the other for the follow-up experiments. When the number of cells reached 1.0 × 10^5^–1.3 × 10^5^ cells/ml, the 0- (control group), 25-, 50-, 100-, and 200-μg/ml BCSC samples were sprayed into each chamber using high-pressure syringes as described above. The cells were then incubated further at 37 °C under 5% CO_2_ and 95% humidity conditions for 24 h. In addition, we cultured HPAEpiC in a 96-well plate using the conventional method (submerged condition) as a comparison for the ALI–DC platform. Briefly, 120 μl of the cell solution (1 × 10^6^ cells/ml) was added to each well. When the number of cells reached 1.2 × 10^5^–1.5 × 10^5^ cells/ml, the cells were exposed to the same BCSC concentrations using the atomization method described above. Finally, the ALI–DC platform and the 96-well plate were placed in a humidified incubator at 37 °C and 5% CO_2_ for 24 h. In submerged exposures, DMEM containing different concentrations of BCSCs (0, 25, 50, 100, and 200 μg/ml) were directly added to each well of the plate.

### Cellular staining with fluorescent dyes

ROS production and cell apoptosis were measured using flow cytometry. Details can be found in the Supplementary Information “Additional file 1: Methods.”

### Dynamic culture optimization

To optimize the dynamic culture flow rate, HPAEpiC were cultured on the ALI–DC platform with medium flow rates of 10, 20, 30, 40, and 50 μl/min, respectively. Cells were cultured in the 96-well plates (submerged condition) for comparison. Cell viabilities were measured at 6, 12, 24, 48, and 72 h. Briefly, at the end of each experiment, the medium was removed from the platform by washing with PBS solution. After washing, 0.25% trypsin solution was added to the PC membrane surfaces to harvest cells. The cell viability was measured using a Trypan Blue Staining Cell Viability Assay Kit according to the manufacturer’s instructions.

### Determination of cell viability and apoptosis rate after exposure to BCSCs

After exposure to BCSCs at concentrations of 0 (control), 25, 50, 100, and 200 μg/ml for 24 h, cells on the ALI–DC platform and 96-well plates were collected into flow cytometry tubes. The cell viabilities and apoptosis rate were measured following the method mentioned in the Supplementary Information “Additional file 1: Methods.”

### Scanning electron microscopy

To observe the deposition and morphology of particles, a SEM was used to compare the BCSC particles on sampling membranes and the ALI–DC platform. All the membrane specimens were fixed on the sample stage of the SEM, respectively, and treated with spray-gold prior to observation. Samples were then imaged with a Mira3 LM (Tescan, Czech Republic) SEM, using a secondary electron detector.

### Transmission electron microscopy

After treatment with BCSCs at a concentration of 200 μg/ml for 24 h, the cells were digested with 0.25% trypsin according to the method described above before being harvested into EP tubes. After centrifugation, the cells were fixed with 2.5% glutaraldehyde at 4 °C for 24 h. Subsequently, the cells were washed with PBS solution three times and postfixed with 1% osmium tetroxide for 2 h at 25 °C. The cells were then dehydrated in graded ethanol solutions, infiltrated with epoxide resin, and cured at 60 °C for 48 h. Finally, the cells were cut into ultrathin Sects. (40–70 nm) using a FC7-UC7 (Leica, Germany), stained with 1% uranyl acetate and 0.1% lead citrate for 30 min each, and ultimately observed under a Tecnai G220 TWIN transmission electron microscope (TEM; FEI, America).

### Western blot analysis

After exposure to BCSC solutions of 0, 25, 50, 100, and 200 μg/ml for 24 h, the protein expression levels were detected using western blotting. Details can be found in the Supplementary Information “Additional file 1: Methods.”

### Statistical analyses

Data are presented as the mean ± standard error of the mean, deriving from three individual experiments (n = 3) unless otherwise stated. Statistical analyses were performed using SPSS software (SPSS Inc., USA). Multiple comparisons of data were conducted using one-way ANOVA, and mean differences between two groups were tested using independent Student’s t-tests. P-values less than 0.05 were considered statistically significant.

## Supplementary Information

**Additional file 1**: The chemicals and reagents, cell viability assay, apoptosis assay, detection of mitochondrial ROS, endotoxin measurement by ELISA, and western blot analysis are described in the Supplementary Material. In addition, three results are discussed in the Supplementary Material.

## Data Availability

All data and materials are specifically referenced within the manuscript. These are all openly available.

## Abbreviations

HPAEpiC: Human pulmonary alveolar epithelial cells
ALI: Air–liquid-interface
DC: Dynamic culture
BCSCs: Biomass combustion soluble constituents
TEM: Transmission electron microscope
SEM: Scanning electron microscope
PC: Polycarbonate
ROS: Reactive oxygen species
NAC: *N*-Acetyl-cysteine
PM_2.5_: Particulate matter 2.5
PDMS: Polydimethylsiloxane
FBS: Fetal bovine serum
DMEM: Dulbecco’s modified Eagle’s medium
PBS: Phosphate-buffered saline
FITC: Fluorescein isothiocyanate
PI: Propidium iodide
DAPI: 4′,6-Diamidino-2-phenylindole-2-dihydrochloride
PVDF: Polyvinylidene difluoride
BSA: Bovine serum albumin
PMSF: Phenylmethanesulfonyl fluoride
TBST: Tris-buffered saline, 0.1% Tween-20
SDS-PAGE: Sodium dodecyl sulfate polyacrylamide gel electrophoresis
