# Supplementary material for: Cytotoxicity analysis of biomass combustion particles in human pulmonary alveolar epithelial cells on an air–liquid interface/dynamic culture platform
Source: Part Fibre Toxicol. 2021 Aug 21;18:31. doi: 10.1186/s12989-021-00426-x (PMC8379799; doi:10.1186/s12989-021-00426-x)
Supplement: Supplementary file 1 — Additional file 1: The chemicals and reagents, cell viability assay, apoptosis assay, detection of mitochondrial ROS, endotoxin measurement by ELISA, and western blot analysis are described in the Supplementary Material. In addition, three results are discussed in the Supplementary Material. [file 12989_2021_426_MOESM1_ESM.docx]

**Supplementary Information**

**Cytotoxicity analysis of biomass combustion particles in human** **pulmonary alveolar epithelial cells on an air–liquid interface/dynamic culture platform**

**Shaorui Ke^1,^** **^2^,** **Qi Liu^2^, Xinlian** **Zhang^2^, Yuhan Yao^2^, Xudong Yang*^,3^, Guodong Sui*^,2,4^**

^1^ Co-construction Collaborative Innovation Center for Chinese Medicine and Respiratory Diseases by Henan & Education Ministry of P.R. China, Academy of Chinese Medical Sciences, Henan University of Chinese Medicine, Zhengzhou 450046, China

^2^ Shanghai Key Laboratory of Atmospheric Particle Pollution Prevention (LAP3), Department of Environmental Science & Engineering, Fudan University, Shanghai 200433, P.R. China

^3^ Department of Building Science, Tsinghua University, Beijing 100084, P.R. China

^4^ Jiangsu Collaborative Innovation Center of Atmospheric Environment and Equipment Technology (CICAEET), Nanjing University of Information Science & Technology, Nanjing 210044, P.R. China

E-mail: Shaorui Ke: [15110740019@fudan.edu.cn](mailto:15110740019@fudan.edu.cn);

Qi Liu: [13110740002@fudan.edu.cn](mailto:13110740002@fudan.edu.cn);

Xinlian Zhang: [15210740031@fudan.edu.cn](mailto:15210740031@fudan.edu.cn);

Yuhan Yao: 16110740002@fudan.edu.cn;

Xudong Yang: [xyang@tsinghua.edu.cn](mailto:xyang@tsinghua.edu.cn);

Guodong Sui: [gsui@fudan.edu.cn](mailto:gsui@fudan.edu.cn).

**Methods and Materials**

**Chemicals and reagents**

Human pulmonary alveolar epithelial cells (HPAEpiC) were obtained from the Shanghai Institute of Cellular Resources, Chinese Academy of Sciences, China. Fetal bovine serum (FBS), 0.25% trypsin-EDTA solution, dulbecco's modified eagle's medium (DMEM) with high glucose, penicillin/streptomycin solution, and phosphate buffered saline (PBS) were purchased from Thermo Fisher Scientific, USA. ROS Detection Assay Kit, Antioxidants N-acetyl-cysteine (NAC), Tubulin Tracker Red, Enhanced BCA Protein Assay Kit, Trypan Blue Staining Cell Viability Assay Kit, Annexin V-Fluorescein Isothiocyanate (FITC) Apoptosis Detection Kit, Radio Immunoprecipitation Assay (RIPA) lysis buffer and 4′, 6-Diamidino-2-phenylindole-2-dihydrochloride (DAPI) were purchased from Beyotime Biotechnology, China. Bacterial Endotoxin ELISA Kit was obtained from Shanghai Enzyme-linked Biotechnology, China. Polydimethylsiloxane (PDMS) was acquired from Momentive Specialty Chemicals, USA. PC membranes with 3 μm pore size were obtained from Whatman, England. All antibodies were procured from Abcam, Hong Kong. Other reagents were purchased from Sigma-Aldrich, USA.

**Cell viability assay**

The cells on the platform were stimulated with BCSCs at concentrations of 0, 50, 100, 150, 200 μg/mL and incubated for 24 h, respectively. At the end of the exposure experiment, 0.25% trypsin solution was simultaneously injected into each chamber f for incubation 3 min, then the cells on the surface of PC membranes were collected. Cell viability was determined with trypan blue assay.

**Apoptosis assay**

To measure the apoptosis rate, the collected cells were stained with fluorescein isothiocyanate (FITC) and propidium iodide (PI) and detected using an Annexin V-FITC Apoptosis Detection Kit according to the manufacturer's instructions. FITC and PI were excited using a 488-nm laser and detected under 525 nm and 620 nm wavelengths, respectively. The apoptosis rate was directly determined using a flow cytometer (Gallios, Beckman Coulter, America).

**Detection of** **mitochondrial ROS**

HPAEpiC were collected after the exposure to BCSCs at concentrations of 0, 25, 50, 100, and 200 μg/ml for 24 h, respectively. Subsequently the specimens were re-suspended in 5 μM of MitoSOX solution and incubated in the dark for 15 min at 37°C. After the staining, the treated cells were washed three times with PBS to remove extra MitoSOX probes, finally re-suspended in 200 μl of PBS, and immediately measured with the flow cytometer. Fluorescence was excited with a 488 nm beam, and emissions were obtained at a wavelength of 525 nm.

**Endotoxin measurement by ELISA**

For quantitative standard curve plotting, solution of endotoxin (ET) standards was prepared at final concentrations of 0.01, 0.02, 0.04, 0.08 and 0.16 ng/ml and then tested by the Bacterial Endotoxin ELISA Kit. The color change of each specimen was analyzed by a Microplate Reader at 450 nm (Multiskan FC, Thermo, USA). The standard curve was plotted by taking the concentrations of the ET as the X-axis, and the OD value as the Y-axis. Then, the ET level in 1-mg/ml BCSCs stock solution was determined using the standard curve.

In order to verify whether levels of ET in BCSCs solutions had impacts on the growth of HPAEpiC, a series concentration of ET solutions (0.025, 0.5, 0.75 and 1 ng/ml) were prepared to cover the concentration of ET in the BCSCs (25-200 μg/ml). Then, the ET solutions of 60 μl were sprayed on the ALI carrying HPAEpiC for 24-h exposures, respectively. Finally, the cell viabilities were tested following the method mentioned above.

**W****estern blot analysis**

The cells were harvested and lysed in cold RIPA lysis buffer with 1mM of phenylmethanesulfonyl fluoride (PMSF) for 5 min. The supernatants containing proteins were collected for western blotting analysis after centrifugation at 14000 rpm for 3 min. The total protein content of each group was determined using an enhanced BCA Protein Assay Kit. Protein samples were denatured at 100 °C for 10 min, separated on 12% sodium dodecyl sulfate polyacrylamide gel electrophoresis (SDS-PAGE) gels, and transferred to polyvinylidene difluoride (PVDF) membranes. The PVDF membranes were blocked with 5% bovine serum albumin (BSA) in PBST buffered for 2 h at room temperature and incubated with primary antibody solutions in Tris-buffered saline, 0.1% Tween-20 (TBST) at 4 °C overnight. Subsequently, the membranes were washed with PBST buffer three times and incubated with secondary antibody solutions for 2 h at room temperature. The immunoreactive bands were developed with the ECL reagents and visualized after exposure of the X-ray film. The band density of the target proteins was standardized according to that of β-actin.

**Results**

## Detection of endotoxin concentrations in BCSC solution

The standard curve of endotoxin (ET) is shown in Fig. S1. ET concentrations in the range of 0.01 to 0.16 ng/ml had a good linear relationship with the OD value. The R^2^ was >0.99, demonstrating that the standard curve can be used to quantify ET in the samples. Thus, the ET concentration in the BCSC test solution (20-fold dilution from the 1 mg/ml stock solution) was approximately 0.11 ± 0.03 ng/ml. Therefore, the ET levels in BCSC solutions of 25, 50, 100, and 200 μg/ml were approximately 0.06, 0.11, 0.22, and 0.44 ng/ml, respectively. To verify whether such levels of ET would impact HPAEpiC growth, HPAEpiC cells were exposed to ET solutions at concentrations of 0, 0.025, 0.5, 0.75, and 1 ng/ml under ALI-DC conditions. As shown in Fig. S1B, the cell viabilities were between 92% and 96% after treatment with ET for 24 h. No significant effects on HPAEpiC growth were observed, even at ET concentrations much higher than those in the BCSC samples.

**
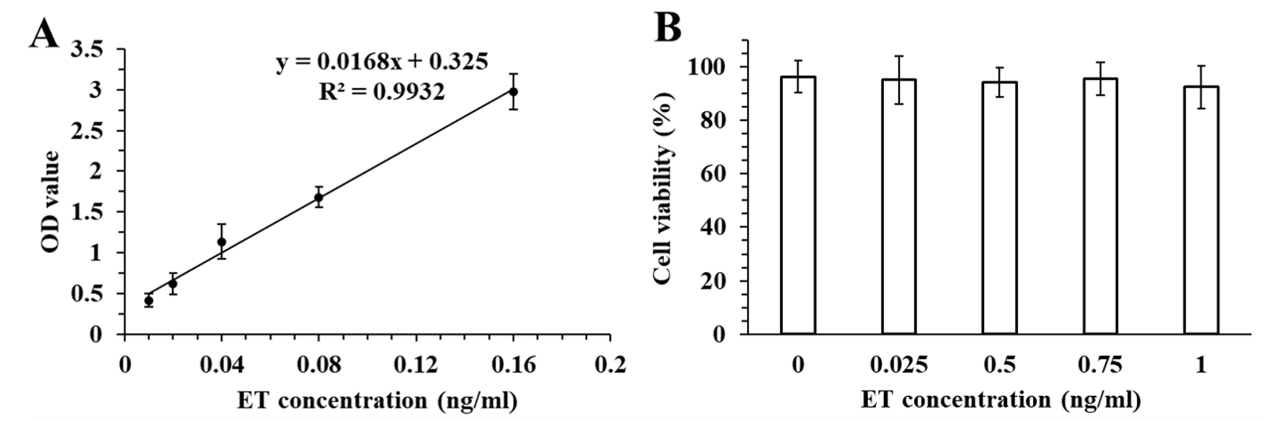
**

**Fig. S1** (A) Standard curve for the quantitative endotoxin (ET) test; (B) human pulmonary alveolar epithelial cell viabilities after 24-h exposures to different concentrations of ET on the air–liquid.interface–dynamic culture platform.

***Mitochondrial ROS generation after HPAEpiC exposure to BCSCs***

Compared with the 0 μg/ml BCSCs control group, the proportion of positive peaks remarkably increased with increasing BCSC concentrations (Fig. S2). The mitochondrial ROS increased more than 30% after treatment with 25 μg/ml BCSCs, increasing by 80% in the 200 μg/ml BCSC treatment. ROS generation was associated with BCSC concentration in a dose-dependent manner.


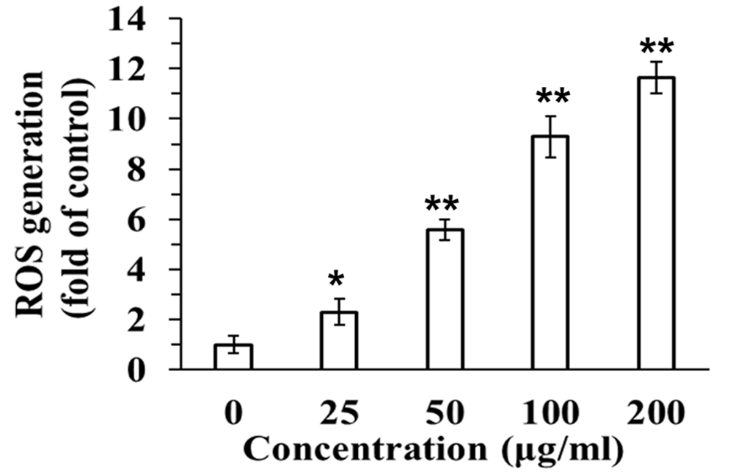


**Fig. S2** Reactive oxygen species (ROS) generation in human pulmonary alveolar epithelial cells exposed to 0–200 μg/ml biomass combustion soluble constituents (BCSCs). Percentage of intracellular ROS expressed as average ± standard deviation of three independent experiments (* *P* < 0.05, ** *P* < 0.01).

***Protective effects of NAC against BCSC-induced cytotoxicity in HPAEpiC***

After HPAEpiC exposure to 200 μg/ml BCSCs, the ROS production, phosphorylated p53 expression, and apoptosis rate increased considerably, while cell viability decreased significantly compared with those in the control group (Fig. S3). However, after adding 5-mM N-acetyl cysteine (NAC), the mitochondrial ROS generation was reduced by two-thirds, phosphorylated p53 expression was reduced by more than one-third, and the apoptotic rate decreased by nearly half. In addition, significant differences in the cell viability, phosphorylated p53 expression, ROS production, and apoptosis rate were observed between the NAC-added group and the control group.


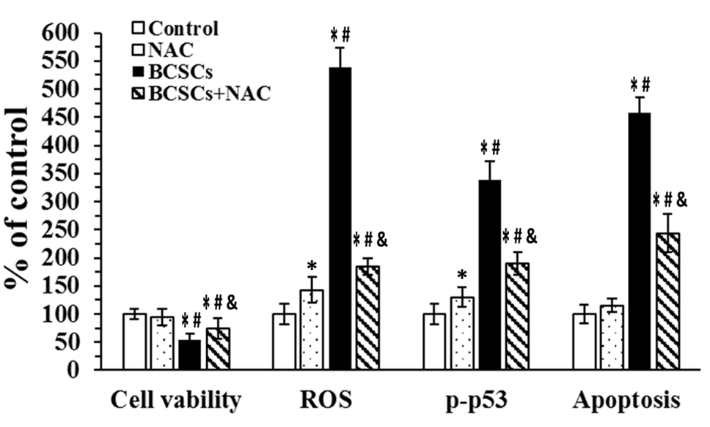


**Fig. S3** Human pulmonary alveolar epithelial cells exposed to 200 μg/ml biomass combustion soluble constituents (BCSCs) and N-acetyl cysteine (NAC) simultaneously showed a higher cell viability and lower reactive oxygen species (ROS) production, phosphorylated p53 expression, and apoptosis rate than cells exposed to BCSCs alone (* *P* < 0.05, compared with the control group values; # *P* < 0.05, compared with NAC group values; & *P* < 0.05, compared with BCSC group values).
